# Editorial: Technology-assisted cultural diversity learning

**DOI:** 10.3389/fpsyg.2023.1274292

**Published:** 2023-08-31

**Authors:** Rustam Shadiev, Wu-Yuin Hwang, Fahriye Altinay, David Guralnick

**Affiliations:** ^1^College of Education, Zhejiang University, Hangzhou, China; ^2^College of Science and Engineering, National Dong Hwa University, Shoufeng, Taiwan; ^3^Societal Research and Development Center, Faculty of Education, Institute of Graduate Studies, Near East University, Nicosia, Cyprus; ^4^Teachers College, Kaleidoscope Learning and Columbia University, New York, NY, United States

**Keywords:** technology-assistive/supportive, cultural diversity, learning, instruction, Editorial

In today's interconnected and multicultural world, cultural diversity learning holds significant importance (Durko and Martens, 2021). With increasing globalization and interdependence among people (Shadiev et al., 2021), it becomes essential to equip individuals with relevant knowledge, skills, and competences to thrive in diverse environments (Stefanova and Jiménez, 2019). As a result, many countries have integrated cultural diversity learning at all academic levels, encompassing multicultural, intercultural, cross-cultural, and intra-cultural learning, among other forms of cultural education. This inclusive approach incorporates cultural diversity learning into various contexts such as education, business, healthcare, tourism, and more (Shadiev and Dang, 2022). Learners in these settings not only grasp their primary academic subjects but also acquire essential cultural knowledge and skills (Imara and Altinay, 2021).

Technology plays a pivotal role in supporting various initiatives (Hwang et al., 2022). It facilitates the learning of academic subjects and cultural diversity (Shadiev and Dang, 2022). For instance, tele-collaboration and virtual exchange learning projects have been implemented, allowing representatives from diverse cultures to engage without the need for extensive travel (Barbosa and Ferreira-Lopes, 2021). Through messaging, video conferencing, and virtual reality technologies, learners can immerse themselves in cultural learning experiences, fostering better cross-cultural understanding and competence. Technology has also been applied in areas like medical and global health education, where learners from different countries can interact online to exchange ideas and discuss culture-related topics (Wu et al., 2021). In the context of informatics, learners have created virtual panoramic tours showcasing local cultural attractions, enabling creativity, innovation, and entrepreneurship (Shadiev et al., 2022).

However, most studies have primarily focused on technology-assisted cultural diversity learning within language learning contexts and classroom activities (Bahari, 2022), leaving other learning contexts and activities outside the classroom relatively unexplored by educators and researchers (Shadiev and Dang, 2022). To address this gap, researchers are urged to consider inclusiveness in various learning contexts and conditions, emphasizing the importance of technology in managing diversities effectively. To shape global citizens and meet the needs of a rapidly changing world, educators must pay attention to educational, technological, intracultural, intercultural, and cross-cultural aspects. This necessitates the proposal of innovative ideas related to technology-assisted cultural diversity learning, encompassing various theories, approaches, techniques, methods, and processes.

The aim of this Research Topic is to gather pioneering theoretical work and original applications concerning technology-assisted cultural diversity learning in diverse learning contexts. The research focus on learning models and theories that elucidate this crucial dimension, exploring their applications in cultural diversity learning within different learning environments and demonstrating their effectiveness through systematic or empirical data.

The Research Topic comprises the following six articles:

Kwee and Dos Santos study explores integrating digitized heritage buildings into blended ESL teaching to enhance students' achievement of Sustainable Development Goals and promote cultural awareness. Researchers used social cognitive theory and a case study approach, revealing themes like expanding knowledge beyond the curriculum and incorporating cultural heritage in language learning. Cultural elements and Sustainable Development Goals positively influenced language acquisition, technology use, and interdisciplinary knowledge.

Bahçelerli study investigates the impact of innovative technology on multicultural vocational education. With 36 students enrolled in tourism education, the study uses qualitative approach and action research methodology, employing semi-structured interviews for data collection. The findings highlight positive views on technology's significance in fostering market growth and access, and students' awareness of advantages and disadvantages in online multicultural vocational education, emphasizing technology's importance in the tourism industry.

Lai study explores integrating UAV technology into culturally responsive teaching. The research aims to establish a UAV-assisted teaching environment for multi-ethnic students, engaging in problem-solving with computational thinking while programming UAVs. Culturally responsive teaching fosters understanding and cooperation among students from diverse backgrounds, enhancing learning effectiveness and cultural respect. The method improves programming skills and deepens comprehension of various cultures in a multicultural education context.

Yu and Wu study examines EFL university students' perceptions of cross-cultural videoconferencing presentations for US professors. Nineteen English-as-foreign-language students participated, responding positively to the activity. They appreciated the meaningful use of English, motivation to prepare thoroughly, and authentic interaction. Concerns included language proficiency, anxiety, and limited preparation time. Nevertheless, they highlighted the profound impact of the presentation experience.

Zort et al. study investigates sharing cultural values through storytelling in digital environments. Experts' perspectives on utilizing the digital platform for cultural value sharing are examined. The study emphasizes the crucial role of educators and families in this process. Findings reveal concerns about cultural value erosion, underscoring the importance of preservation and transmission to younger generations. Active participation in digital platforms, especially with community-oriented and human-centered computing approaches, facilitates effective cultural heritage projects.

Rai et al. study analyzes MOOCs fostering intercultural competence through language learning. It identified relevant keywords, target languages, offering countries, and embedded soft skills. “Culture” was the dominant keyword in language teaching MOOCs, highlighting its link to intercultural communication. The study found a limited number of languages and offering countries, while emphasizing the importance of soft skills like language learning, communication, business, entrepreneurship, career development, and cultural understanding in intercultural competence within language education.

## Author contributions

RS: Conceptualization, Methodology, Supervision, Writing—original draft, Writing—review and editing. W-YH: Conceptualization, Methodology, Supervision, Writing—review and editing. FA: Conceptualization, Methodology, Supervision, Writing—review and editing. DG: Conceptualization, Methodology, Supervision, Writing—review and editing.

## References

[B1] Bahari A. (2022). Affordances and challenges of technology-assisted language learning for motivation: A systematic review. Inter. Learn. Environ. 20, 248. 10.1080/10494820.2021.2021246

[B2] Barbosa M. W. Ferreira-Lopes L. (2021). Emerging trends in telecollaboration and virtual exchange: A bibliometric study. Educ. Rev. 75, 558–586. 10.1080/00131911.2021.1907314

[B3] Durko A. Martens H. (2021). Fostering higher level cultural learning among tourism students through virtual interaction. J. Teach. Travel Tour. 21, 235–247. 10.1080/15313220.2021.1880350

[B4] Hwang W. Y Guo B. C. Hoang A. Chang C. C. Wu N.T. (2022). Facilitating authentic contextual EFL speaking and conversation with smart mechanisms and investigating its influence on learning achievements. Comput. Assist. Lang. Learn. 9, 406. 10.1080/09588221.2022.2095406

[B5] Imara K. Altinay F. (2021). Integrating education for sustainable development competencies in teacher education. Sustainability 13, 12555. 10.3390/su132212555

[B6] Shadiev R. Dang C. (2022). A systematic review study on integrating technology-assisted intercultural learning in various learning context. Educ. Inf. Technol. 27, 6753–6785. 10.1007/s10639-021-10877-6

[B7] Shadiev R. Wang X. Y. Wu T. T. Huang Y. M. (2021). Review of research on technology-supported cross-cultural learning. Sustainability 13, 1402. 10.3390/su13031402

[B8] Shadiev R. Yi S. Dang C. Sintawati W. (2022). Facilitating students' creativity, innovation and entrepreneurship in a telecollaborative project. Front. Psychol. 13, 887620. 10.3389/fpsyg.2022.887620 35572324 PMC9100410

[B9] Stefanova A. Jiménez A. G. (2019). Building bridges between cultures–an experiment with collaborative online international learning (COIL). J. Teach. English Spec. Acad. Purp. 6, 535–542. 10.22190/JTESAP1803535S

[B10] Wu A. Maddula V. Singh J. Sagoo M. G. Chien C. L. Wingate R.. (2021). Alternatives to student outbound mobility—Improving students' cultural competency skills online to improve Global Health without travel. Med. Sci. Educ. 31, 1441–1451. 10.1007/s40670-021-01332-9 34123512 PMC8184130

